# Utility of Dynamic ^68^Ga-DAZA-PET/CT for Bile Leak Localization After Liver Transplantation: First Clinical Experiences

**DOI:** 10.3390/biomedicines14010022

**Published:** 2025-12-22

**Authors:** Anke Werner, Oliver Rohland, Julia Greiser, Martin Freesmeyer, Utz Settmacher, Robert Drescher, Felix Dondorf

**Affiliations:** 1 Department of Nuclear Medicine, Jena University Hospital, 07747 Jena, Germany; anke.werner@med.uni-jena.de (A.W.); julia.greiser@med.uni-jena.de (J.G.); robert.drescher@med.uni-jena.de (R.D.); 2 Department of General, Visceral, and Vascular Surgery, Jena University Hospital, 07747 Jena, Germany; oliver.rohland@med.uni-jena.de (O.R.); utz.settmacher@med.uni-jena.de (U.S.); felix.dondorf@med.uni-jena.de (F.D.)

**Keywords:** liver transplantation, bile leak, hepatobiliary imaging, HCC, DAZA, PET/CT

## Abstract

**Background/Objectives**: Biliary complications are common after liver transplantation (LT), with bile leaks representing a major cause of morbidity. Conventional imaging modalities such as ultrasound, CT, MRCP, and endoscopic techniques may fail to localize peripheral or complex leaks. This study aimed to evaluate the feasibility of [^68^Ga]Ga-TEoS-DAZA-PET/CT for non-invasive localization of bile leaks after LT. **Methods**: Five male patients (mean age 53.2 years) with suspected bile leakage and inconclusive prior imaging underwent [^68^Ga]Ga-TEoS-DAZA-PET/CT. The tracer was synthesized under GMP conditions and administered at a mean activity of 204 ± 42 MBq. Dynamic PET/CT imaging was performed for 60 min, and findings were classified according to the Nagano classification. **Results**: Bile leaks were detected and anatomically localized in all five patients. Sites included the liver resection surface, central bile ducts, bilioenteric anastomosis, and biliary drainage exit. PET/CT findings guided revision surgery in one case and endoscopic treatment in three, while one patient improved without intervention. No adverse effects occurred. **Conclusions**: [^68^Ga]Ga-TEoS-DAZA-PET/CT is a feasible and safe imaging technique for the anatomical localization of bile leaks following LT. Its antegrade visualization of biliary flow, high spatial and temporal resolution, and lack of contraindications make it a promising complementary modality when conventional imaging is inconclusive or not feasible. Larger studies are warranted to validate its diagnostic value and clinical utility in postoperative and post-traumatic biliary injuries.

## 1. Introduction

Biliary complications are the most common adverse events following liver transplantation (LT), occurring in approximately 5–32% of recipients and representing a major cause of morbidity [1]. Post-transplant complications are classified as early (within 4 weeks) or late, with bile leaks being the most frequent. Postoperative diagnostic imaging plays a crucial role in early detection, localization, and characterization of bile leaks. In a step-up approach, sonography, contrast-enhanced CT, magnetic resonance cholangiopancreatography (MRCP), endoscopic retrograde cholangiography (ERC), and percutaneous transhepatic cholangiography (PTC) may be performed for anatomical depiction of the bile ducts, depending on the clinical situation, potential therapeutic approach, and availability [1]. The Nagano classification categorizes postoperative bile leaks following partial liver resection and is also applicable after LT, particularly in cases of split grafts. It classifies leaks according to their anatomical origin within the biliary tree, providing guidance for selecting the appropriate therapeutic approach [2]. When these diagnostics are unsuccessful or contraindicated, hepatobiliary scintigraphy (HBS) can be used to locate and quantify the bile leaks [3]. However, the spatial and temporal resolution of scintigraphic images is limited compared to positron emission tomography/computed tomography (PET/CT).

The radiotracer, [^68^Ga]Ga-tri-ethoxysalicyl-1,4-diazepan-6-amine ([^68^Ga]Ga-TEoS-DAZA), has recently become available for hepatobiliary function and bile duct imaging. It is a lipophilic radiotracer that demonstrates rapid hepatocellular uptake followed by biliary excretion. The radiopharmaceutical can be synthesized and quality-controlled under Good Manufacturing Practice (GMP) conditions by labeling gallium-68 with the TEoS-DAZA precursor [4]. Recently, a toxicity study of the TEoS-DAZA precursor was performed, confirming the pharmacological safety of the compound [5]. Our analysis aimed to evaluate the applicability of this tracer for the PET-based localization of bile leaks in the setting of LT.

## 2. Materials and Methods

Patients with clinical suspicion of bile leakage, persistent biliary drainage in the postoperative setting of LT, and previous inconclusive or insufficient imaging were examined. The precursor was acquired from Inflamed Pharma GmbH, Jena, Germany. All patients provided written informed consent for the conduction of [^68^Ga]Ga-TEoS-DAZA-PET/CT (DAZA-PET/CT). The research was conducted in accordance with both the Declarations of Helsinki and Istanbul and was approved by our ethics committee (Reg. no. 2025-3873-BO-D).

The radiotracer [^68^Ga]Ga-TEoS-DAZA is a lipophilic complex of neutral net charge that is rapidly taken up by hepatocytes and is subsequently excreted into the bile [4,6]. The synthesis and quality control of the radiopharmaceutical for clinical application can be performed in accordance with GMP by labeling gallium-68 with TEoS-DAZA precursor in HEPES buffer on an automated synthesizer (Scintomics GRP 3V, Scintomics att, Fuerstenfeldbruck, Germany). Post-labeling purification via SPE cartridge (SEP-PAK C18 light, Waters, Eschborn, Germany) with ethanol in a two-step procedure (1. 0.7 mL ethanol 40% *v*/*v*, 2. 1.0–2.0 mL ethanol 50% *v*/*v*) is important to remove both HEPES and undesired by-products. Radiochemical purity (RCP) was determined by testing for non-labeled gallium-68 by radio thin layer chromatography (TLC) with 0.1 M sodium citrate on silica gel 60 plates and by testing for colloidal gallium-68 with glass fiber TLC plates (ITLC-SG) and a mixture (1/1 *v*/*v*) of methanol and ammonium acetate (77 g/L) as eluent. Additionally, RCP was determined by radio high-performance liquid chromatography (HPLC) using a C18 reversed-phase column (length 125 mm, diameter 4 mm) and the following gradient: 0.0–2.5 min 97.0% A, 2.5–10.0 min 97.0% A → 0.0% A, 10.0–13.0 min 0.0% A, 13.0–13.05 min 0.0% A → 97.0% A, 13.05–16.0 min 97.0% A (A: water/trifluoroacetic acid (99.9/0.1 *v*/*v*), B: acetonitrile/trifluoroacetic acid (99.9/0.1 *v*/*v*). RCP of all radiotracer batches was ≥95.0%.

Recently, a toxicity study of the TEoS-DAZA precursor was performed, confirming the pharmacological safety of the compound [5]. [^68^Ga]Ga-TEoS-DAZA was administered to patients with a mean activity of 204 ± 42 MBq (range, 144–256 MBq). The mean and standard deviation of the administered mass of TEoS-DAZA precursor was 40.5 ± 15.1 µg (range, 21.1–78.3 µg).

The patients were positioned supine on a Biograph Vision PET scanner (Siemens Healthineers, Erlangen, Germany). For attenuation correction, an unenhanced low-dose CT scan (50 mAs; 120 kV) of the upper abdomen was performed. Early dynamic PET was conducted in list mode, beginning simultaneously with the intravenous administration of the radiotracer bolus (3 MBq per kg body weight, minimum 150 MBq) and continuing for 60 min. Image reconstruction was performed with the TrueX HD algorithm (OSEM with PSF modelling, implemented in syngo PET scanner reconstruction software, version VG.80, Siemens Healthineers, Erlangen, Germany) with 3D attenuation-weighted ordered subsets and expectation maximization at four iterations, 12 subsets with a 5 mm post-reconstruction Gaussian filter, attenuation image segmentation, and a 512 × 512 pixel matrix. Image review was performed with syngo.via Oncology module (Siemens Healthineers, Erlangen, Germany). The findings were classified according to Nagano’s classification system.

## 3. Results

**Figure 1:**
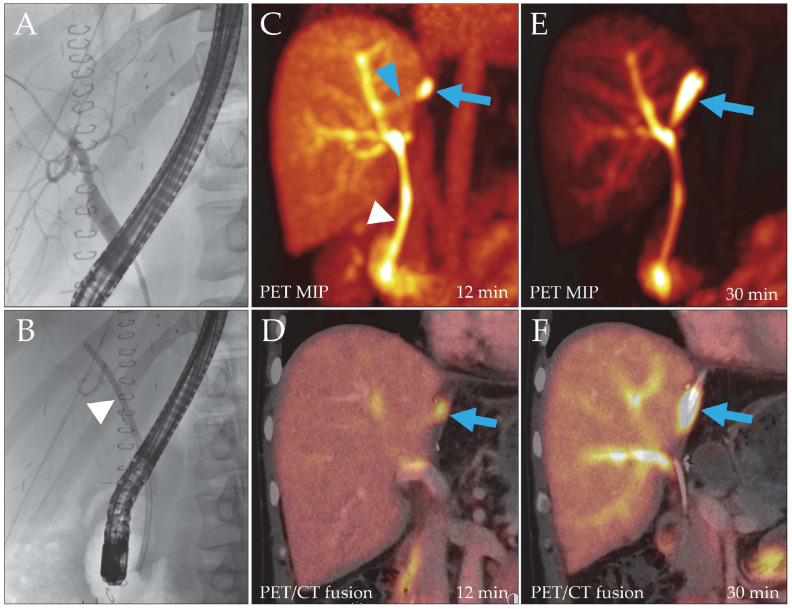
Patient 2, liver recipient. Post-transplantation ERC (**A**) did not reveal a bile leak, but to facilitate biliary drainage, a hepatoenteric stent was placed ((**B**), arrowhead). Due to persistent drainage of bile through the catheter, a DAZA-PET/CT was performed. Despite a patent stent ((**C**), white arrowhead), it showed a leakage site at the liver resection surface from 12 min after tracer injection ((**C**,**D**), blue arrow; SUVmax 29.7/SUVmax/mean liver tissue 9.9/7.8) draining into a bilioma, which filled over time ((**E**,**F**), blue arrows). A small bile duct branch appeared to reach the leak from the main right hepatic ducts ((**C**), blue arrowhead). MIP, maximum intensity projection.

**Figure 2:**
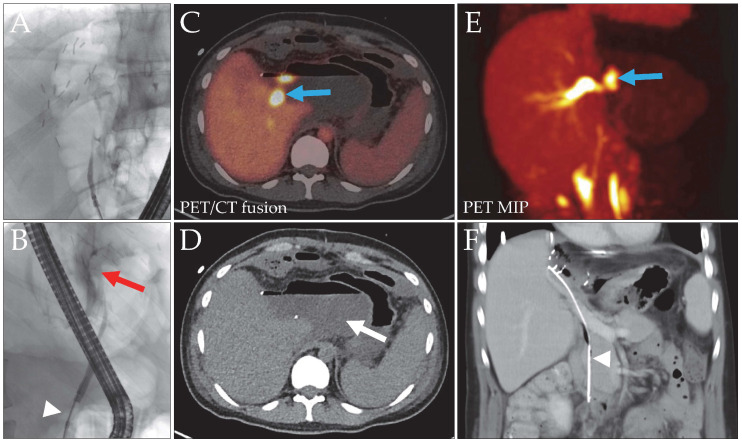
Patient 3, liver donor, with a bile leak after left hemihepatectomy for LDLT. The initial ERC image (**A**) did not show the biliary tree. With a blocked catheter ((**B**), white arrowhead), a contrast media extravasation (red arrow) without clear leak localization was evident. Intervention with stent placement was not possible due to the stricture. DAZA-PET (**C**,**E**) revealed a central bile leak (blue arrows, SUVmax 43.0/SUVmax/mean liver tissue 9.4/7.0), draining into a large bilioma ((**D**), arrow). The space-occupying bilioma caused external compression of the central bile ducts, resulting in distention of the right intralobar ducts. After bilioma and leak repair surgery, a long stent was implanted to facilitate bile outflow ((**F**), arrowhead). MIP, maximum intensity projection.

The study period spanned from June 2024 to April 2025, during which 70 liver transplantations were performed. All patients with clinical suspicion of post-transplant bile leakage were referred to PET/CT by transplant surgeons. The patient cohort included five men with a mean age of 53.2 years (range 26.1–72.2 years) (Table 1). Four patients were LT recipients, and one patient was a left lobe living donor. No adverse or clinically detectable pharmacological effects were observed in any patient. Prior imaging included ERC in four patients and biliary drain fluoroscopy in one patient. It was positive for bile leak detection in three of the five cases, but in two cases, with no clear localization. DAZA-PET/CT could detect (3 patients) or further localize (2 patients) suspected bile leakage. Bile leaks were identified at the liver resection surface (Figure 1, Supplementary Video S1), at the central bile ducts (Figure 2, Supplementary Video S2), at the bilioenteric anastomosis, and at the exit site of the biliary drainage in one case, respectively. The immediate therapeutic consequences were revision surgery in one patient and endoscopic intervention in three patients. No further intervention was needed in a patient with spontaneous decline in leakage. One patient later underwent retransplantation for hepatic artery thrombosis. All patients are alive.

## 4. Discussion

This study aimed to investigate the feasibility of DAZA-PET/CT in patients after deceased-donor or living-donor LT and evidence of biliary tract insufficiency.

To date, no PET tracer for functional liver imaging has been approved for routine clinical use. The recently developed radiotracer [^68^Ga]Ga-TEoS-DAZA can be synthesized under GMP conditions and has been implemented for hepatobiliary function imaging and appears particularly useful to depict, and potentially quantify, the excretory liver function [4,7]. Biliary tree evaluation is not possible with the oncologic tracers [18F]FDG, [18F]F-/[68Ga]Ga-PSMA and [68Ga]-DOTATOC tracers because they are not biliary excreted. Moreover, [^68^Ga]Ga-TEoS-DAZA-PET/CT shows a safe toxicity profile [4,5]. In its first human application in a patient with suspected bile duct obstruction, biliary excretion was visualized along the entire common bile duct [7]. Subsequent use in a patient with traumatic liver injury demonstrated a bile leak, with [^68^Ga]Ga-TEoS-DAZA-PET/CT revealing focal tracer accumulation at the inferior surface of the left hepatic lobe, later confirmed intraoperatively as a laceration. The whole-body radiation effective dose resulting from the administration of 150 MBq (4.05 mCi) of gallium-68 to an adult weighing 75 kg is about 3.15 mSv. The low-dose abdominal CT scan for attenuation correction accounts for approximately 0.8–2 mSv [8]. Determination of the organ dose to the liver requires further evaluation.

In our study, bile leaks were detected and anatomically correlated in all five patients. In four patients, therapeutic interventions were performed based on PET/CT findings. The PET protocol for this indication comprises successive dynamic PET acquisitions of 20 min following the attenuation-correction CT of the upper abdomen. PET data are reconstructed into 60 s frames. As each acquisition finishes, image reconstruction and preliminary evaluation can begin while the next acquisition is still underway. This permits real-time image assessment during the scan, ideally with the surgical team. If no leak is detected after 60 min, further 20 min acquisitions may be performed. It may also be beneficial to have the patient stand and move around briefly before additional imaging. Another option to be evaluated is the use of respiratory-gated PET acquisition, which increases image clarity but decreases temporal resolution.

Quick and anatomically accurate diagnosis of biliary complications after LT facilitates the selection of the most suitable treatment strategy and improves clinical outcomes. The precise detection of bile leaks is of critical clinical importance. In orthotopic liver transplantation, bile leaks most commonly arise from the biliary anastomosis—the well-recognized “Achilles heel” of the procedure. In contrast, the situation in living donor liver transplantation is more complex. In addition to central bile leaks related to anastomotic insufficiency, peripheral bile leaks may also occur. Moreover, isolated or disconnected bile ducts can be present, representing an even greater therapeutic challenge. Differentiating between these entities is essential for appropriate treatment planning, particularly in newly immunosuppressed patients. In this context, the described novel functional diagnostic method has the potential to substantially influence clinical decision-making.

Recent hepatobiliary imaging studies indicate that no standardized algorithmic approach currently exists for the diagnosis of biliary leaks. CT is frequently associated with non-specific findings, scintigraphy is time-consuming and limited by low spatial resolution, and MRCP represents the primary modality for evaluation of the biliary tree [9]. ERC and PTC enable simultaneous therapeutic intervention but carry a risk of serious procedure-related complications [10]. DAZA-PET/CT may help close the diagnostic gap by visualizing bile ducts that show limited accessibility by endoscopy, especially peripheral bile leaks. The key advantage of DAZA-PET/CT over ERC lies in its different imaging mechanisms. ERC generates projection images through a retrograde approach—injecting contrast medium under pressure against the natural flow of bile. DAZA-PET/CT visualizes the biliary system in an antegrade manner, aligning with the physiological direction of bile flow. Antegrade imaging can provide a more comprehensive depiction of biliary anatomy and function, potentially revealing pathologies that may be overlooked by retrograde techniques. In a retrospective study assessing the diagnostic accuracy of MRCP compared with PTC and ERC, MRCP was shown to reliably identify the presence of bile leakage in patients with low serum bilirubin levels [10]. Furthermore, a recent review describes contrast-enhanced MRCP as the current “gold standard” for biliary imaging, enabling highly accurate detection and localization of bile leaks [11]. Nevertheless, in comparison with MRCP, PET/CT allows better dynamic visualization of biliary flow and is more robust against motion and metal artifacts [12]. PET(/CT) images can be displayed in both the cross-sectional and 3D views.

As mentioned above, several techniques for hepatobiliary imaging have been established. Despite its role as a first-line imaging modality, MRCP sometimes proves inconclusive in clinical practice, as image quality can be compromised, for example, by motion-related artifacts. Given that MRCP visualizes fluid based on T2-weighted contrast rather than bile per se, its interpretability may be reduced in complex postoperative cases, as bilomas and other fluid accumulations can be indistinguishable from native bile ducts. Given their invasive nature, ERC and PTC can be helpful for patients requiring therapeutic intervention [13]. ERC is inherently limited in assessing extra-biliary structures, leading to a high false-negative rate due to the inability to visualize ducts disconnected from the main biliary system [14,15]. Additionally, ERC is rarely feasible in patients who have undergone pancreaticoduodenectomy (classic Whipple’s operation) or pylorus-preserving pancreatoduodenectomy with reconstruction [16]. PTC is effective in confirming and precisely identifying a bile leak, but it is even more invasive than ERC and is associated with potential complications. These include the risk of infection leading to cholangitis, cholecystitis, abscess, peritonitis, and sepsis, as well as non-infectious complications like bile duct injuries and severe hemorrhage [17]. Moreover, certain methods may not be appropriate for all patients due to anatomical factors and contraindications, such as allergies to contrast agents. Because only minimal tracer quantities are required to generate a detectable signal, nuclear medicine imaging procedures generally have no contraindications, apart from pregnancy.

Based on these considerations, [^68^Ga]Ga-TEoS-DAZA-PET/CT may represent a valuable diagnostic option for patients with suspected bile leakage of unclear origin or localization, providing non-invasive anatomical information that may assist therapeutic treatment planning.

Limitations of our study are the small sample size of 5 patients and the monocentric experience. Although promising, our findings require validation in larger patient cohorts. Moreover, availability of PET/CT is limited to larger centers. It is also important to acknowledge the radiation exposure associated with PET/CT imaging; however, given the clinical context and the severity of disease in the patient population under consideration, this factor is generally of limited concern. False-positive cases were not observed in our cohort, but it is possible that an aberrant bile duct may be misinterpreted as a small bile leak, particularly in complex postoperative settings. To date, no tracer accumulations outside the hepatobiliary and renal systems have been observed.

## 5. Conclusions

[^68^Ga]Ga-TEoS-DAZA-PET/CT demonstrates potential as a non-invasive imaging modality for the precise anatomical localization of bile leaks after LT, particularly in complex postoperative or post-traumatic cases, where it may close diagnostic gaps between established imaging techniques. Its physiological antegrade imaging mechanism and combination of high temporal and spatial resolution facilitate the detection of leaks beyond the reach of ERC procedures. Based on the limited experience from small case series, DAZA-PET may be a valuable clinical tool in patients with suspected bile leakage of unclear origin or localization, as it enables precise, non-invasive anatomical characterization of biliary injury and facilitates individualized treatment planning.

## Figures and Tables

**Table 1:** Clinical and procedural characteristics.

Patient No.	Age	Diagnosis	Surgical Procedure	Indication for DAZA-PET/CT	Bile Leakage Site	Nagano Classification	Treatment Decision
1	72.2	Ischemic biliary lesions after LT for hepatocellular carcinoma in ethyl-toxic liver cirrhosis	LT recipient	Persistent bile leakage and liver abscesses due to ITBL and HAT	Leakage at exit site of the biliary drainage (Roder drainage)	A	ERC/stent, retransplantation after 134 days
2 (Figure 1)	38.7	Intrahepatic cholangiocarcinoma	LDLT recipient (right lobe)	Persistent bile leakage 45 days postoperatively	Liver resection surface	A	None (leakage declining)
3 (Figure 2)	26.1	Left lobe donor for LDLT	LDLT donor (left lobe)	Persistent bile leakage 16 days postoperatively	central bile ducts	D	re-laparotomy, bilioma evacuation, ERC with stent for hilar stenosis
4	61.2	Hepatocellular carcinoma in ethyl-toxic liver cirrhosis	LT recipient	Persistent bile leakage after conversion of duct-to-duct anastomosis to BEA	leakage of the BEA	D	PTCD
5	67.6	Colorectal liver metastases	LT recipient	Persistent bile leakage 30 days postoperatively	Liver resection surface	A	ERC/stent

BEA, bilioenteric anastomosis; ERC, endoscopic retrograde cholangiography; LDLT, living donor liver transplantation; LT, (orthotopic) liver transplantation; HAT, hepatic artery thrombosis; PTCD, percutaneous transhepatic cholangiodrainage.

## Data Availability

The data presented in this study are available on request from the corresponding author due to privacy restrictions.

## Supplementary Materials

The following supporting information can be downloaded at https://www.mdpi.com/article/10.3390/biomedicines14010022/s1, Supplementary Video S1: Supplement 1_patient 2.mp4; Supplementary Video S2: Supplement 2_patient 3.mp4.

## Abbreviations

The following abbreviations are used in this manuscript:

BEA	bilioenteric anastomosis
ERC	endoscopic retrograde cholangiography
GMP	Good Manufacturing Practice
HAT	hepatic artery thrombosis
ITBL	ischemic type biliary lesions
HBS	hepatobiliary scintigraphy
LDLT	living donor liver transplantation
LT	liver transplantation
MIP	maximum intensity projection
MRCP	magnetic resonance cholangiopancreatography
PET/CT	positron emission tomography/computed tomography
PTC	percutaneous transhepatic cholangiography
PTCD	percutaneous transhepatic cholangiodrainage
TEoS-DAZA	tri-ethoxysalicyl-1,4-diazepan-6-amine
